# Evaluation of Different Strategies to Incorporate Absolute Abundance into Habitat Selection Modeling for the Endangered *Cheirotonus jansoni*

**DOI:** 10.3390/insects17060537

**Published:** 2026-05-22

**Authors:** Tianwu Ma, Rui Guo, Qian Lei, Zhangfeng Cheng, Qingyun Wang, Qiao Li, Hong Wu, Junhao Huang, Liwei Liu

**Affiliations:** 1 State Key Laboratory of Climate System Prediction and Risk Management, Nanjing Normal University, Nanjing 210023, China; 2 Jiangsu Center for Collaborative Innovation in Geographical Information Resource Development and Application, Nanjing 210023, China; 3 School of Environment, Nanjing Normal University, Nanjing 210023, China; 4 Administration of Zhejiang Qingliangfeng National Nature Reserve, Hangzhou 311300, China; 5 College of Forestry and Biotechnology, Zhejiang A&F University, Hangzhou 311300, China; 6 Jingning She Autonomous County Ecological Forestry Development Center, Lishui 323500, China; 7 Zhejiang Museum of Natural History, Hangzhou 310014, China

**Keywords:** *Cheirotonus jansoni*, species distribution model, cost-sensitive learning, habitat suitability, sampling effort, potential distribution, GeoAI

## Abstract

Developing habitat selection models is crucial for predicting the potential distribution of rare and endangered insects; however, current modeling approaches largely ignore absolute abundance data from sampling records. Integrating absolute abundance as sample weights involves two contrasting ecological hypotheses: one assumes high abundance reflects superior habitat quality (direct weighting), while the other attributes high abundance to spatially biased sampling effort (inverse weighting). Both hypotheses are plausible for *Cheirotonus jansoni* (Jordan, 1898), a highly phototactic endangered species, complicating the selection of an appropriate weighting strategy. This study evaluated four weighting strategies (unweighted, linear, inverse, and inverse square) derived from these hypotheses. Results indicated that the sampling effort correction strategy (inverse weighting) outperformed habitat-quality-driven strategies (linear weighting) in habitat suitability mapping, achieving a higher AUC (93.41) compared to the linear weighting (91.32). Notably, the linear weighting strategy performed worse than the unweighted baseline, yielding a lower AUC (91.32) than the baseline’s 93.11. These findings provide methodological guidance for modeling the distribution of phototactic insects and offer scientific support for the in situ conservation, ex situ site selection, and supplementary field surveys of *C. jansoni*.

## Simple Summary

Insect surveys record not only where a species is found but also the number of individuals caught at each site. These counts can be used to assign importance to different locations when creating maps that predict where a species is likely to live. Currently, there are two conflicting ideas on how to use this information: one suggests that high numbers indicate high-quality habitat, while the other suggests that high numbers may simply reflect areas where researchers searched more intensely. For the endangered long-armed scarab beetle (*Cheirotonus jansoni*), both possibilities are plausible. We compared different modeling strategies to determine which approach best predicts the beetle’s distribution. Our results show that correcting for the bias of search intensity leads to significantly more accurate habitat maps than assuming high numbers always equal better habitats. This study provides a more reliable method for identifying critical habitats for rare insects, which is essential for guiding conservation efforts, establishing protected areas, and managing forest ecosystems effectively.

## 1. Introduction

As one of the most species-rich animal groups on Earth, insects play indispensable roles in maintaining ecosystem functions and providing ecological services [1,2,3]. However, their sensitivity to environmental change makes them particularly vulnerable to range contractions [4,5,6,7]. Accurately predicting insect geographic distributions is therefore critical for biodiversity conservation.

Habitat selection models, which establish statistical relationships between species distributions and environmental covariates, are a core methodology in biogeographic research [8,9,10]. These models enable assessment of potential habitats, prediction of distributional dynamics, and guidance for conservation planning. Presence–absence data are the most common input for traditional habitat selection models [11]. However, this binary format overlooks species counts and abundance information inherent in biodiversity surveys [12,13,14]. For instance, raw GBIF records often include numbers of collected individuals and sex ratios—details frequently overlooked during modeling [15]. Recent studies have therefore attempted to integrate count or abundance data into habitat selection modeling frameworks to enhance predictive accuracy without altering model structure [14,16,17].

A primary approach for integrating abundance data into habitat selection models is to incorporate abundance (or species counts) as training sample weights. Existing studies attribute abundance differences among sites to two mechanisms, yielding two distinct weighting methods. The first method directly uses abundance or related metrics as weights, assuming that higher individual counts indicate greater habitat suitability [18]. For example, Yu et al. (2020) [14] implemented this strategy in a boosted regression tree model, weighting presence sites by the normalized product of target species relative abundance and site-level species richness, while uniformly weighting absence sites as 1. This allowed the model to prioritize high-abundance sites during training, emphasizing characteristics of more suitable habitats. Similarly, Conlisk et al. (2022) [16] employed abundance-weighted boosted regression tree but simplified calculations by using only log-transformed absolute abundance of the target species, eliminating the need for community composition data. This demonstrates that even without community-level information, absolute abundance alone can improve habitat selection modeling.

The second method uses the reciprocal of abundance (or species counts) as weights, assuming that high-abundance records largely reflect spatial heterogeneity in sampling effort rather than habitat quality. It compensates for sampling bias by assigning higher weights to low-effort areas, which typically correspond to low-abundance records [19,20,21,22]. For instance, Stolar and Nielsen (2015) [23] validated this method using virtual species simulations, demonstrating that incorporating the reciprocal of sampling effort probability into logistic regression models accurately recovered species distributions, with sensitivity and specificity significantly outperforming uncorrected models. Beever et al. (2025) [24] extended this method to real species by assigning lower weights to spatially proximate samples: two records from the same location each received a weight of 1/2, three records 1/3, and so forth. This down-weighting mitigated prediction biases from uneven spatial sampling effort.

According to the above analysis, differences in abundance or species counts between sites may stem from either habitat quality variation or sampling effort differences, each corresponding to a distinct weighting strategy. However, observational data alone cannot determine the underlying cause, making the choice of weighting strategy uncertain a priori. Consider the rare and endangered *Cheirotonus jansoni* (Jordan, 1898), a Class II National Protected Species in China. Due to habitat loss and narrow distribution, this beetle has experienced a significant population decline, making its conservation a matter of increasing urgency. On the one hand, this species depends on evergreen broad-leaved forests, with larval development requiring thick litter layers and abundant decaying wood [25,26,27]. Here, sites with higher counts may indicate higher-quality habitat, suggesting the first weighting strategy is more appropriate.

On the other hand, as a phototactic insect, *C. jansoni* surveys rely primarily on light trapping [28,29]. Critically, the attraction radius of these traps is highly variable and significantly influenced by vegetation density and canopy cover, which can obstruct light visibility. Consequently, the recorded abundance may be more a reflection of the trap’s local visibility than of the actual population density. Furthermore, this sampling method is constrained by lunar phase, weather, terrain accessibility, and personnel deployment, often result in systematic spatial variation in sampling effort. Areas with high accessibility or sampled on clear, moonless nights typically yield more individuals [30,31], yet may not represent optimal habitat quality. This suggests the second weighting strategy might be more suitable. Therefore, the appropriate weighting strategy for *C. jansoni* habitat selection modeling must be determined through comparative analysis.

This study compares habitat quality-based and sampling effort-corrected weighting strategies in habitat selection modeling for *C. jansoni*. Section 2 introduces the study area, data set, and methodology. Section 3 presents comparative results; Section 4 and Section 5 provide discussion and conclusions, respectively.

## 2. Materials and Methods

### 2.1. Study Area and Data

#### 2.1.1. Study Area

**Figure 1:**
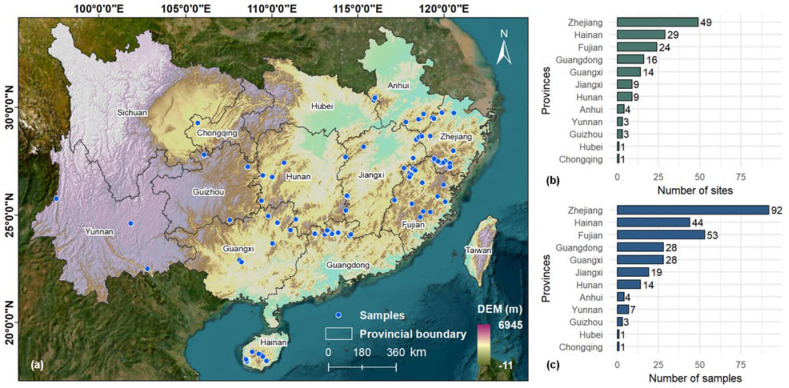
The extent of the study area and the spatial distribution of sampling sites. (**a**) Provinces in China covered by the study area. (**b**) Number of sampling sites in each province. (**c**) Number of samples collected at sampling sites in each province.

This study investigated habitat selection of *C. jansoni* in southern China (Figure 1a). The region spans 20–35° N and 97–122° E, featuring tropical and subtropical climates and topography ranging from plains to plateaus. The study area encompasses 14 provinces, autonomous regions, municipalities, and special administrative regions: Zhejiang, Anhui, Fujian, Jiangxi, Hubei, Hunan, Guangdong, Guangxi, Hainan, Chongqing, Sichuan, Guizhou, Yunnan, and Taiwan.

The study area experiences East Asian monsoon influence and is characterized by a subtropical monsoon climate, with its southern edge extending into the tropical monsoon zone. Annual mean temperature ranges from 15 to 22 °C, with the coldest month generally above 0 °C. Annual precipitation ranges from 800 to 2000 mm, with rainfall and high temperatures coinciding. These hydrothermal conditions support evergreen broad-leaved forests, monsoon forests, and mixed coniferous–broad-leaved forests—the substrate environment upon which *C. jansoni* depends. Primary evergreen broad-leaved forests serve as core habitat; their complex ecosystems, deep litter layers, and abundant decaying wood provide essential food sources and shelter for larval development [26,27]. Key protected areas including Qingliang Peak, Wuyi Mountain, Dinghu Mountain, and Xishuangbanna constitute population refuges for this species.

#### 2.1.2. Sample Collection

To encompass the potential distribution and occurrence of *C. jansoni*, 162 sampling sites were established across southern China based on historical records and structured consultations with regional experts (e.g., specialists from local research institutions and forestry bureaus) (Figure 1b). These experts provided critical insights into the species’ preferred micro-habitats and current land-use statuses, ensuring that the 162 sampling sites were systematically distributed to represent the diverse ecological conditions of southern China [25].

We employed light trapping at each site, following standardized protocols for surveying phototactic beetles. Fieldwork was conducted in phases from April to September to synchronize with geo-graphical variations in adult phenology. Specifically, sampling commenced earlier in southern regions due to higher temperatures and earlier emergence, and was progressively delayed in northern areas along the phenological gradient. Light trapping was conducted nightly from 19:00 to 03:00 the following day to encompass the species’ peak activity period. Surveys were performed biweekly to minimize moonlight interference and optimize personnel allocation. In each province, 2–5 surveyors were deployed, with joint surveys conducted alongside local research institutions to facilitate data sharing on species distribution and abundance. Through systematic repeat surveys, individual counts were recorded to determine absolute abundance at each site (Figure 1c). Necessary collection permits were obtained for Zhejiang Province; in other regions, only non-invasive population monitoring was conducted, with no specimens collected.

#### 2.1.3. Environmental Covariates

As a large saproxylophagous insect, *C. jansoni* exhibits life-stage-specific ecological requirements: larval development is driven by microhabitat-scale thermal metabolism and moisture-dependent decomposition of coarse woody debris, while adult activity is primarily constrained by landscape-scale temperature extremes. To reflect these physiological needs, we constructed a suite of environmental covariates using the WorldClim 2.1 dataset [32] and the NASA SRTM 90 m digital elevation model [33,34]. The climatic variables comprise 19 bioclimatic indicators (30 arc-second resolution, ≈1 km) characterizing annual means, seasonality, and extreme events in temperature and precipitation. Topographic variables were incorporated to account for their influence on species distribution via microclimate modulation and hydrological redistribution.

### 2.2. Construction of Habitat Selection Models

#### 2.2.1. Basic Idea

Traditional habitat selection models typically assign uniform weights to all observation sites, training algorithms on presence–absence data with environmental covariates. However, this framework overlooks the spatial heterogeneity inherent in absolute abundance or individual counts collected during surveys. To address this, we developed a modeling framework that explicitly integrates absolute abundance through two core stages: weight quantification and weighted model training. By generating sample weights based on inter-site variations in abundance, the model applies differential learning intensities during parameter optimization. This approach expands the binary input structure into a ternary input structure (site-covariates-weight). Theoretically, this framework aligns with cost-sensitive learning, a machine learning paradigm that addresses class imbalance or heterogeneous sample quality by penalizing high-cost errors more heavily during the fitting process [35]. We compared several hypothesis-driven weighting strategies to identify the optimal habitat selection modeling for *C. jansoni*.

#### 2.2.2. Quantification of Sample Weights

To incorporate absolute abundance, we developed a baseline and three alternative weighting strategies: unweighted strategy (UW), linear weighting strategy (LW), inverse weighting strategy (IW), and inverse square weighting strategy (ISW). The UW serves as the baseline, assigning a uniform weight of 1 to all sites. This approach follows standard habitat selection modeling protocols by ignoring the spatial variation in absolute abundance.

The LW assumes that abundance is a surrogate for habitat quality, where higher counts indicate more suitable environmental conditions. For presence sites, absolute abundance is directly applied as the weight, prioritizing the environmental characteristics of high-abundance locations during model training; absence sites are assigned a weight of 1.

The IW assumes that high abundance primarily reflects spatial heterogeneity in sampling effort rather than true habitat quality. To mitigate sampling bias, weights for presence sites are calculated as the reciprocal of absolute abundance, granting higher statistical importance to low-abundance records typically associated with lower sampling effort.

The ISW is an extreme variant of the inverse approach, using the inverse square of absolute abundance to penalize high-count sites more severely. This strategy assumes that sampling effort exerts a non-linear amplification effect on abundance where minor increases in intensity (e.g., equipment power or trapping duration) lead to disproportionately high captures. By intensifying the weight reduction, this strategy tests whether aggressive bias correction optimizes model performance. Mathematically, weights are inversely proportional to the square of abundance, maximizing the contribution of low-effort areas while significantly compressing the influence of high-intensity sampling zones.

#### 2.2.3. Integration of Absolute Abundance-Weighted Samples into Random Forests

We employed random forest (RF) as predictive model for habitat selection based on two primary advantages. First, RF facilitates the flexible integration of sample weights into a cost-sensitive learning framework, directly supporting our objective of incorporating absolute abundance. Second, its ensemble learning mechanism effectively suppresses overfitting, ensuring robust performance in habitat selection modeling [36,37].

The RF employs Bootstrap resampling to draw multiple sub-samples with replacement, training an independent decision tree for each. At each node split, a random subset of environmental covariates is evaluated to identify the optimal splitting variable. Individual tree predictions are then aggregated via voting (for classification) or averaging (for regression) to produce the ensemble output [38]. For habitat assessment, we utilized a probabilistic classification mode, using the predicted presence probability as a habitat suitability index (HSI).

Technical implementation is performed using the *ranger* package in R software (4.2.3 version), where weights function during the Bootstrap sampling stage. The probability of a site being selected is proportional to its weight; thus, high-weight sites are sampled more frequently and exert greater influence on model training [39]. This mechanism aligns with cost-sensitive learning, as prediction errors at high-weight sites contribute more heavily to the loss function, forcing the model to prioritize the species–environment associations inherent in those observations. By extending the standard RF workflow into this weighted framework, we integrated absolute abundance data into habitat selection modeling.

### 2.3. Experimental Design

Three experiments were conducted to evaluate the efficacy of the four weighting strategies (UW, LW, IW, and ISW) with respect to predictive performance, weight sensitivity, and spatial extrapolation robustness within the habitat selection modeling.

The first experiment evaluated the direct impact of four weighting strategies on predictive performance. We randomly partitioned the samples into training (60%) and validation (40%) sets, repeating this process over 50 iterations to mitigate stochastic effects. The 1000 background samples are randomly generated across the study area [40,41]. Model performance was assessed on the validation sets using six metrics: area under the curve (AUC), accuracy, precision, recall, F1 score, and the Kappa coefficient [42]. Finally, we trained models using the full dataset to generate habitat suitability map, allowing for a visual comparison of the spatial patterns produced by each weighting strategy.

The second experiment examined the sensitivity of the sample weight. Because absolute abundance may reflect either habitat quality or sampling effort, weights derived from these counts are inherently uncertain. To evaluate robustness, we conducted random weight simulations by drawing 10 sets of weights from two distinct ranges (0–10 and 0–100). The core objective was to determine model responsiveness: significant fluctuations in predictions under randomized weighting would indicate that the model is effectively influenced by the weights, whereas results mirroring the UW baseline would suggest that the weighting exerted no substantial impact.

The third experiment evaluated the spatial extrapolation of weighted models, particularly in data-scarce scenarios. As an endangered species with narrow and fragmented habitats, conservation planning for *C. jansoni* is often constrained by survey data that cover only localized areas, whereas management decisions require a broader spatial scope. To address this, we used Zhejiang Province (characterized by the highest site and individual counts) and Jiangxi Province (representing moderate abundance) as localized training areas. RFs trained on these regions were extrapolated across study area, with data from the remaining provinces used as independent validation sets to assess accuracy.

## 3. Results

### 3.1. Habitat Selection Modeling Using Different Weighting Strategies

**Figure 2:**
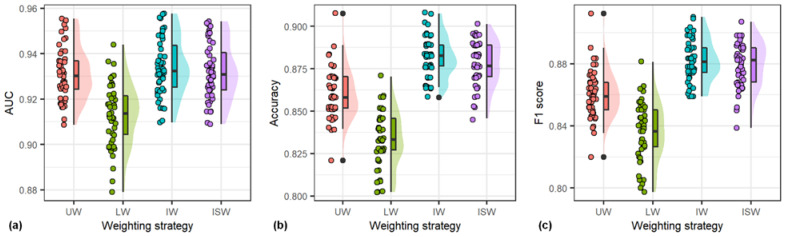
Combination of box plots, violin plots, and dot plots for performance of different weighting strategies for 50 randomly split sample sets. The evaluation metrics are (**a**) area under receiver operating characteristic curve (AUC), (**b**) accuracy, and (**c**) F1 score. UW, LW, IW and ISW represent the unweighted strategy, linear weighting strategy, inverse weighting strategy, and inverse square weighting strategy respectively.

Figure 2 shows the comparative performance of the four strategies across 50 iterations. Overall, IW and ISW outperformed both the UW and LW. The IW emerged as the most robust strategy, followed by ISW, while UW showed intermediate performance; LW consistently yielded the lowest median values and the greatest variability across all metrics. These results suggest that hypotheses based on sampling effort correction enhance predictive accuracy more effectively than those assuming abundance directly reflects habitat quality. Furthermore, excessive penalization of high-abundance sites (ISW) provided no additional benefit and was slightly less effective than moderate correction (IW).

Table 1 summarizes the performance of the four strategies across six metrics: AUC, Kappa, accuracy, precision, recall, and F1-score. Both Table 1 and Figure 2 reveal a distinct performance hierarchy; IW consistently outperformed the other strategies, followed by ISW, UW, and LW. Regarding specific metrics, accuracy and precision followed the sequence IW (88.11%, 87.61%) > ISW (87.79%, 86.60%) > UW (85.96%, 85.61%) > LW (83.28%, 82.04%), demonstrating the advantage of the inverse weighting approach in minimizing false-positive rates. Notably, ISW yielded a slightly higher recall (89.48%) than IW (88.83%), suggesting that while aggressive penalization may identify more potential habitat, it risks introducing false positives. Conversely, the recall for LW (85.23%) was lower than that of the UW baseline (86.51%), confirming that improper weighting can lead to the omission of genuine habitat. As the harmonic mean of precision and recall, the F1-score confirms that IW (88.18%) is the most effective strategy for balancing both types of error. Collectively, the consistency across these six metrics indicates that the IW achieves the optimal balance between suppressing sampling bias and retaining true ecological signals.

**Figure 3:**
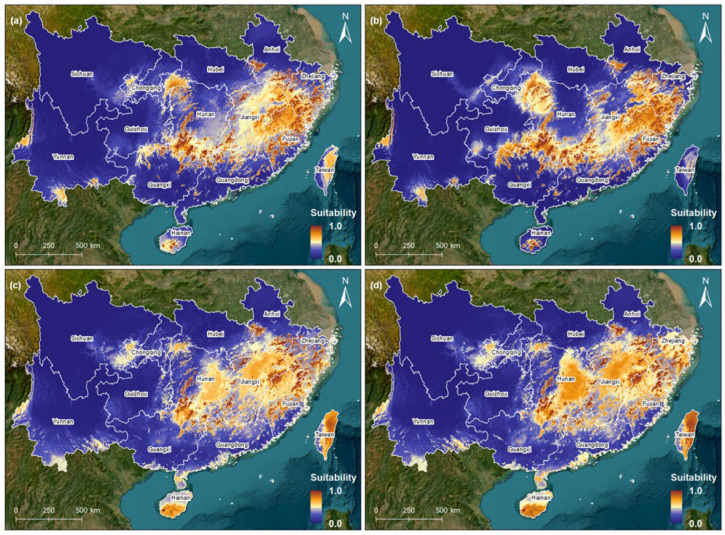
Habitat suitability mapping using different weighting strategies with random forest. (**a**) unweighted strategy, (**b**) linear weighting strategy, (**c**) inverse weighting strategy, and (**d**) inverse square weighting strategy.

Figure 3 shows the habitat suitability maps for *C. jansoni* generated by the four weighting strategies. Overall, the UW and LW (Figure 3a,b) predicted expansive areas of high suitability; however, given that *C. jansoni* is a rare and endangered species characterized by narrow, fragmented habitats, these models likely overestimate its actual range, contradicting empirical field observations. Conversely, the IW and ISW produced more restricted high-suitability distributions, primarily concentrated in mountainous and hilly terrain. These regions, sheltered from significant anthropogenic disturbance, offer the stable microhabitats required for larval development. Notably, Figure 3c,d illustrate that high suitability is not ubiquitous across all mountains, underscoring the species’ specialized ecological niche. This selectivity aligns with its dependence on deep humus and specific coarse woody debris, indicating that distribution is driven by microhabitat quality—such as stand structure and deadwood availability—rather than macro-topography alone. Thus, by mitigating sampling effort bias, the IW and ISW provide a more accurate representation of the species’ true habitat preferences.

### 3.2. Uncertainty from Sample Weights

**Figure 4:**
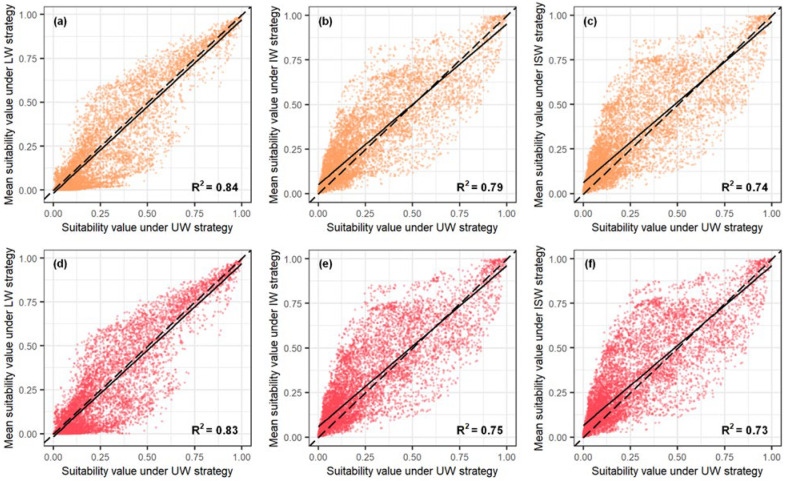
Uncertainty from different weighting strategies. (**a**–**c**) with random weights ranging from 0 to 10, and (**d**–**f**) with random weights ranging from 0 to 100.

Figure 4 illustrates the influence of different weighting strategies on the uncertainty of habitat suitability predictions (Experiment 2). The scatter plots display HSI values from the UW baseline (x-axis) against the mean HSI values derived from 10 stochastic weight perturbations for LW, IW and ISW (y-axis). Figure 4a–c show results for the 0–10 weight range, while Figure 4d–f show perturbations within the 0–100 range.

The LW, IW, and ISW respond differently to weight uncertainty. Across both weight ranges, LW exhibited the highest spatial concordance with the UW baseline (R^2^ = 0.84 for the 0–10 range; R^2^ = 0.83 for the 0–100 range), indicating that LW is relatively insensitive to weight perturbations. In contrast, R^2^ values for the IW (0.79 and 0.75) and ISW (0.74 and 0.73) were lower and their scatter plots were more dispersed. This suggests that sampling effort correction strategies (IW and ISW) are more sensitive to weight configurations. Minor weight variations propagate through the model training process, leading to increased spatial variability in the final suitability assessments.

### 3.3. Spatial Extrapolation

Table 2 shows the spatial extrapolation performance for each weighting strategy, using AUC as the primary metric. Overall, extrapolation accuracy declined significantly compared to the in-sample predictions in Experiment 1, with AUC values dropping from 93% to between 62% and 71%. This decline underscores the inherent predictive uncertainty associated with “local survey, global decision” scenarios. Nevertheless, the relative performance hierarchy remained consistent with Experiment 1, further validating the robustness of the sampling effort correction strategies.

In Zhejiang, the extrapolation AUC for the IW (71.03%) outperformed both UW (66.91%) and LW (63.82%), while ISW (70.10%) followed closely. A consistent trend emerged in Jiangxi (IW: 70.06% > ISW: 69.69% > UW: 65.89% > LW: 62.27%). This indicates that inverse weighting effectively mitigates the spatial propagation of sampling bias, regardless of the abundance levels in the training region. In contrast, the LW leads to bias during extrapolation. Importantly by equating high abundance with high suitability. The performance gap between IW and ISW was minimal (0.93% in Zhejiang and 0.37% in Jiangxi), whereas the difference between UW and IW was substantial (4.12% in Zhejiang and 4.17% in Jiangxi). This suggests that in data-limited extrapolation, the fundamental decision to correct for sampling effort is more consequential.

**Figure 5:**
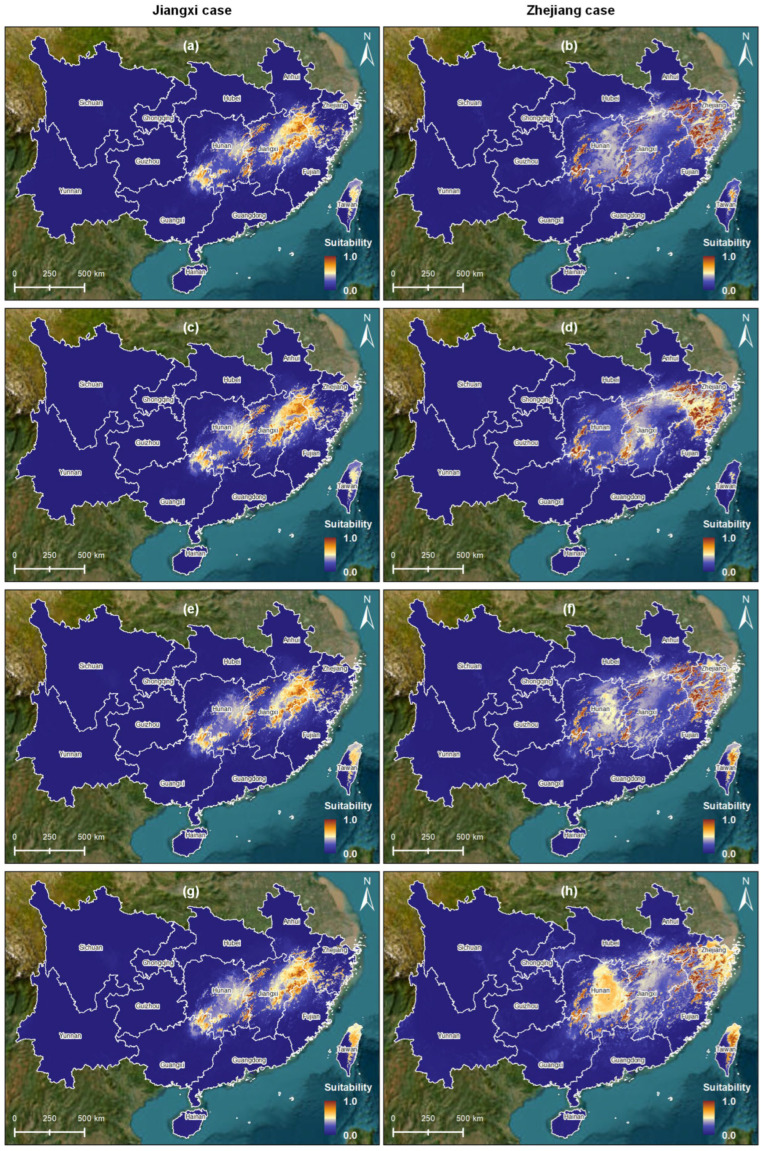
Spatial extrapolation based on the Jiangxi and Zhejiang samples. (**a**,**b**) are the unweighted strategy, (**c**,**d**) are the linear weighting strategy, (**e**,**f**) are the inverse weighting strategy, and (**g**,**h**) are the inverse square weighting strategy.

**Figure 6:**
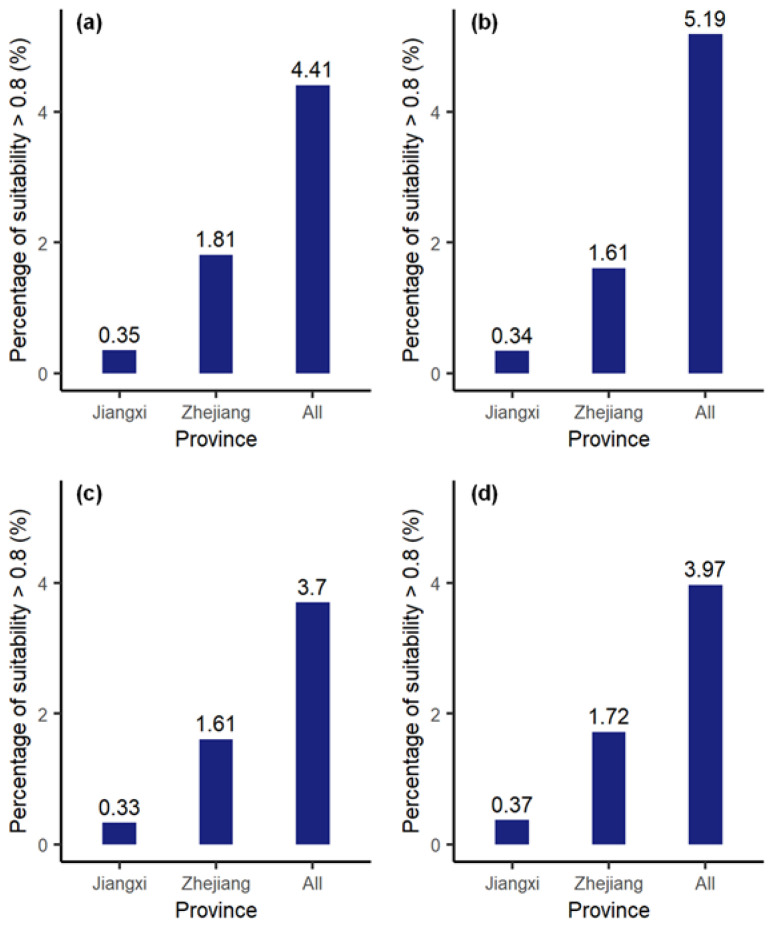
Area ratios of the high suitability zones predicted for the Jiangxi samples, Zhejiang samples, and all samples. (**a**) unweighted strategy, (**b**) linear weighting strategy, (**c**) inverse weighting strategy, and (**d**) inverse square weighting strategy.

Figure 5 illustrates the habitat suitability for *C. jansoni* extrapolated the study area using models trained independently on data from Zhejiang and Jiangxi provinces. Overall, the potential distribution exhibited two primary characteristics. First, high-suitability zones were heavily concentrated near the training localities and along similar latitudinal bands. Second, compared to the full-dataset results in Figure 3, the extrapolated suitable range was significantly more restricted, with high-suitability patches showing increased fragmentation. These findings suggest that while *C. jansoni* is distributed across Southern China, it maintains highly specialized habitat requirements. Its population persistence relies on specific environmental configurations, leading to conservative model predictions when encountering environmental novelty. While minimizing false positives provides a “safety mechanism” for prioritizing certain habitats, such conservative predictions risk missing cryptic populations in unsurveyed regions. This trade-off underscores the importance of ground-truthing in predicted low-to-moderate suitability zones. Figure 6 compares the proportions of predicted high-suitability areas (HSI > 0.8) across the four strategies using models trained on Jiangxi, Zhejiang, and the full dataset. Proportions derived from the two provincial (local) datasets were significantly lower than those from the full-sample models, corroborating the conservative nature of localized predictions. Specifically, under the UW, high-suitability habitat accounted for only 0.35% in the Jiangxi model and 1.81% in the Zhejiang model, compared to 4.41% for the full model (Figure 6a). The LW further compressed these proportions to 0.34% and 1.61%, respectively (Figure 6b). Similar patterns were observed for the IW and ISW (Figure 6c,d).

## 4. Discussion

### 4.1. Impact of Absent Sample Weights

**Figure 7:**
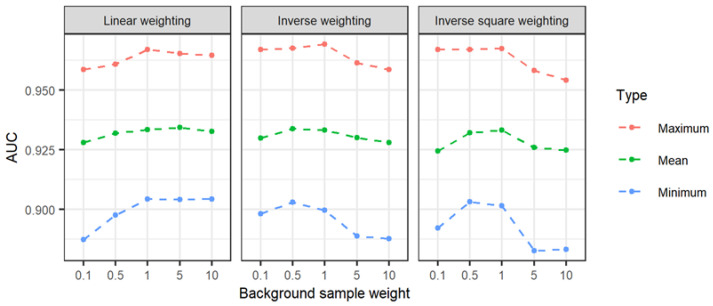
The relationship between background sample weights and prediction accuracy in weighted strategies.

Weights assigned to background samples were also integrated into the model training process. Building on Experiment 1, we varied background weights set from 0.1 to 10 to evaluate their impact on the performance of the three weighting strategies (Figure 7). The results indicated that background weight variations had a limited influence on mean AUC, with all strategies maintaining high performance within the 0.5~1.0 range. However, the impact on minimum AUC was more pronounced; weights between 5 and 10 led to a marked decline in the minimum AUC. This suggests that excessive weighting of background samples increases the risk of performance fluctuations and compromises predictive robustness.

### 4.2. Applications and Limitations

The superior performance of the inverse weighting strategies (IW and ISW) is directly tied to the light-trapping characteristics of *C. jansoni*. Because trap placement often depends on expert knowledge and site accessibility, sampling hotspots frequently emerge in easily reachable areas [43,44,45], confounding absolute abundance with sampling effort. These conclusions are likely generalizable to other phototactic insects or taxa where survey designs are primarily experience-driven.

We recommend evaluating sampling design characteristics before implementing absolute abundance weighting. If site selection is significantly influenced by subjective experience or convenience, inverse weighting should be prioritized to suppress spatial bias. Conversely, linear weighting may be appropriate for systematic grid-based surveys with uniform coverage. For abundance data sourced from public repositories (e.g., GBIF), researchers should conduct preliminary assessments to compare the performance of LW and IW before finalizing the weighting scheme.

This study has several limitations. First, our analysis focused on a single species using absolute abundance as the weighting criterion. In community-level research, relative abundance (e.g., the proportion of a target species within the total community) may be more appropriate, as it can partially standardize interspecific differences in sampling effort. Previous research suggests that relative abundance is less sensitive to sampling intensity than absolute abundance, offering greater robustness for cross-site comparisons [14]. Future studies should explore the distinct contexts in which absolute versus relative abundance weighting strategies are most effective multispecies assemblages.

Second, our modeling was restricted to the RF. While it features a flexible weighting interface, other machine learning models also support cost-sensitive learning. For instance, XGBoost allows for explicit sample weight adjustments [46,47], and contemporary deep learning architectures utilizing attention mechanisms can adaptively learn site importance weights [48]. Since different algorithms exhibit varying response mechanisms to weighting, cross-model comparisons represent a valuable direction for future study.

Third, future assessments should aim for finer-scale modeling to bridge the gap between global climate data and microhabitat requirements. Incorporating UAV-based vegetation mapping and high-resolution topographic indices at the site or protected-area scale will allow for a more nuanced representation of the micro-environmental factors that influence the species’ life cycle.

### 4.3. Habitat Conservation

Field observations suggest that *C. jansoni* selectively inhabits environments adjacent to large trees, primarily Fagaceae species. These slow-growing hardwood trees provide a stable, long-term food source for larvae, whose developmental period spans two years or more. Consequently, the availability of coarse woody debris is a critical limiting factor. The spatial patterns of habitat suitability identified in this study corroborate these ecological requirements; highly suitable areas are predominantly concentrated in mid-to-subalpine zones, exhibiting high-altitude, fragmented, and restricted distributions (Figure 3) that mirror the range of large Fagaceae trees. This spatial pattern is highly consistent with the climate suitability zones predicted by Yu and Li (2024) [26] based on climatic factors. Yu and Li (2024) [26] similarly found that the potential distribution of *C. jansoni* is mainly concentrated in mountainous forest areas at 1000–2000 m elevation in southeastern China, such as the Nanling and Wuyi Mountains.

Accordingly, in situ conservation for *C. jansoni* should prioritize the protection and restoration of high-mountain forests, with a focus on maintaining the natural accumulation of coarse woody debris and prohibiting the removal of deadwood. Furthermore, our habitat suitability maps provide a scientific framework for ex situ conservation and population management. By identifying high-suitability areas where occurrences have not yet been recorded, conservationists can prioritize supplementary surveys and potential reintroductions. Finally, these maps offer a guide for optimizing future sampling efforts; establishing new survey sites in remote, high-suitability, but data-poor mountainous regions will effectively fill existing data gaps and enhance the spatial representativeness of long-term monitoring programs.

## 5. Conclusions

This study builds a habitat selection modeling framework explicitly integrating absolute abundance data and compares two hypothesis-driven weighting strategies for *C. jansoni*. Our findings indicate the following: (1) correction for sampling effort is superior to assuming abundance reflects habitat quality, as IW and ISW outperformed the unweighted baseline, while LW proved least effective; (2) moderate bias correction is more effective than excessive penalization, with IW providing the optimal balance between suppressing sampling artifacts and retaining ecological signals; and (3) in data-constrained extrapolation scenarios, IW and ISW effectively mitigate the spatial propagation of bias from sampling hotspots. The resulting habitat suitability maps offer a scientific foundation for conservation planning and the optimization of future surveys. This framework is generalizable to other phototactic insects.

## Figures and Tables

**Table 1:** Performance comparison of habitat selection modeling based on different weighting strategies.

Weighting Strategy	Evaluation Metric (%)					
	AUC	Kappa	Accuracy	Precision	Recall	F1 Score
Unweighted strategy (UW)	93.11	71.92	85.96	85.61	86.51	86.02
Linear weighting strategy (LW)	91.32	66.56	83.28	82.04	85.23	83.57
Inverse weighting strategy (IW)	93.41	76.22	88.11	87.61	88.83	88.18
Inverse square weighting strategy (ISW)	93.21	75.58	87.79	86.60	89.48	87.79

**Table 2:** Performance of spatial extrapolation for habitat selection modeling based on different weighting strategies trained in Zhejiang and Jiangxi provinces.

Weighting Strategy	AUC (%)	
	Zhejiang	Jiangxi
Unweighted strategy (UW)	66.91	65.89
Linear weighting strategy (LW)	63.82	62.27
Inverse weighting strategy (IW)	71.03	70.06
Inverse square weighting strategy (ISW)	70.10	69.69

## Data Availability

The original contributions presented in the study are included in the article; further inquiries can be directed to the corresponding author.

## Abbreviations

The following abbreviations are used in this manuscript:

RFs	Random forests
HSI	Habitat suitability index
UW	Unweighted strategy
LW	Linear weighting strategy
IW	Inverse weighting strategy
ISW	Inverse square weighting strategy
